# Acknowledgement to Reviewers of *Nutrients* in 2014

**DOI:** 10.3390/nu7010349

**Published:** 2015-01-07

**Authors:** Nutrients Editorial Office

**Affiliations:** MDPI AG, Klybeckstrasse 64, CH-4057 Basel, Switzerland

The editors of *Nutrients* would like to express their sincere gratitude to the following reviewers for assessing manuscripts in 2014:

Abdelmalek, Manal F.
Abe, Sumiko
Abenavoli, Ludovico
Abner, Erin
Adelman, Daniel C.
Adjé, Félix
Adlard, Paul
Agho, Kingsley E
Aguilera-Tejero, Escolástico
Ahmed, Serge
Aisbett, Brad
Alam, Samina
Alda, Martin
Al-Dujaili, Emad
Alex, Sheril
Alexander, Szalai,
Alissa, Eman M.
Allen, Jason D.
Allott, Emma
Allsopp, Philip
Althuis, Michelle
Alwan, Na
Alway, Stephen E.
Amin, Rajesh
Andersen, Jens Rikardt
Andersen, Vibeke
Anderson, Alex Kojo
Anderson, John
Andersson, Helena
Andrade, Juan
Andrade, Paula Andrade
Andreoli, Angela
Angeloni, Cristina
Angulo-Barroso, R.M.
Annabi, Borhane
Annunziata, Azzurra
Anstey, Nick
Antonio, Joey
Aoi, Wataru
Apalset, Ellen M.
Apone, Fabio
Appleton, Katherine
Armanini, Decio
Armenta, Roberto
Armitage, Andrew E.
Arora, Pankaj
Arroo, Randolph
Aruoma, Okezie
Asard, Han
Aselage, Melissa B
Ashour, Mohamed
Atlantis, Evan
Augustin, Livia
Aukema, Harold M.
Aura, Anna-Marja
Auricchio, Salvatore
Austin, Gregory
Averna, M.R.
Azcona, Cristina
Baba, E.
Babatunde, Oyinlola
Bailey, Stephen
Bailo, Bibiana Garcia
Baker, Julien
Baker, Robert
Bakovic, Marica
Baldwin, Christine
Banu, Jameela
Baragetti, andrea
Barbagallo, Mario
Barbarossa, Iole Tomassini
Barclay, Alan W
Barker, Tyler
Barnett, Matthew P.G.
Barocelli, Elisabetta
Baron, Kelly Glazer
Barreira, João
Barton, Elisabeth R.
Barvencik, Florian
Battino, Maurizio
Baumgartner, Jeannine
Beatty, Stephen
Beaven, C. Martyn
Beitz, Donald C.
Bellisle, France
Belski, Regina
BEMBEN, Michael G.
Bentley, David
Berendschot, Tos
Berge, Kjetil
Berger, Nathan A.
Berglund, Staffan
Berning, Jacqueline R.
Bertoia, Monica L.
Betts, Jonathan W.
Bhatia, Jatinder
Bielohuby, Maximilian
Birch, Edward
Bizzaro, Nicola
Bjorklund, Anneli
Bjornstad, Petter
Blanton, Cynthia
Blindauer, Claudia A.
Blusztajn, Jan
Boaz, Mona
Bogdanski, Pawel
Bogl, L.H.
Boisson-Vidal, Catherine
Booth, Alison
Borg, Søren
Borghi, Claudio
Börnhorst, Claudia
Bortolus, Renata
Bosy-Westphal, Anja
Boutsikou, Theodora
Bozzetti, Federico
Bozzetto, L.
Brabham, Brian
Bradbury, Kathryn E.
Braidy, Nady
Bramanti, Emilia
Brandhagen, Martin
Brand-Miller, Jennie C.
Brantsaeter, Anne
Braun, Barry
Bravi, Francesca
Breidenassel, Christina
Breit, Samuel N.
Bright, John J.
Brinkman, Maree T.
Brodribb, Wendy
Brophy, James M.
Brotherton, Carol S.
Brough, Louise
Brown, Andrew W
Brown, Jonathan
Brown, Lindsay
Brown, Rachel
Bryan, Nathan S.
Bryant, Eleanor J.
Bryszewska, Maria
Buchowski, Maciej S.
Buckland, Genieve
Buechler, Christa
Burdge, Graham
Burgess, John R.
Burmeister, Jacob
Burns, David
Buscemi, Silvio
Byrn, Mary
Cai, Yan
Calabrò, S.
Calder, Philip C.
Callaghan-Koru, Jennifer A.
Calvo, Mona
Campa, Adriana
Campbell, Norm R.C.
Campion, Bruno
Canas, Atilio
Candow, Darren
Canto, Carlos
Capewell, Simon
Carlberg, Carsten
Carless, Melanie A.
Carmel, Ralph
Caroli, Margherita
Carr, Anitra C.
Casanovas, Carmen
Cass, Wayne A.
Castaño, Angélica
Castaño, Paula M.
Castelao, Alberto Martinez
Castleman, Tony
Cederbaum, Arthur
Cermak, R.
Cervellati, Carlo
Cetinkaya, Ali
Chachay, Veronique S.
Chalmers, Kerry
Chan, Dick
Chan, K.K.L.
Chan, Maria
Chan, Queenie
Chan, Ruth
Chan, Yong Shin
Chand, Sourabh
Chang, Matt
Chaumontet, Catherine
Chelland Campbell, Sara
Chen, Chung-Yen
Chen, Chung-Yi
Chen, Ming-Hsuan
Chen, Ruei-ming
Chen, Tong
Chen, Yun
Chen, Zhe-Sheng
Cheng, HL
Cherniack, E. Paul
Childs, Emma
Chilibeck, Phil
Chilton, Robert J.
Chitchumroonchokchai, Julie
Chiu, Ken C.
Chmurzynska, Agata
Cho, Youn-Ok
Christaki, Efterpi
Christopher, Gary
Chu, Fong Fong
Chun, Ock
Chung, Jay H.
Ciaccio, Edward J.
Ciancarelli, Maria Giuliana Tozzi
Ciccone, Marco Matteo
Cicero, Arrigo F.G.
Cieślik-Boczula, Katarzyna
Clarke, Gerard
Claudia, Vetrani
Clemens, Roger
Coan, P.M.
Coates, Alison M.
Cobiac, Linda
Coe, Christopher L.
Coëffier, Moïse
Cohen, Ronald
Colby, Sarah
Collier, D.N.
Collins, Clare
Collins, James F.
Conlon, Beth A.
Conlon, Cath
Conrad, Kirk
Considine, Robert V.
Conterno, Lorenza
Conti, Ario
Cook, Brian
Cook, Nancy R.
Cooke, Matt
Corazza, Gino Roberto
Corbin, Karen
Cordero, Paul
Cordero-Macintyre, Zaida R.
Corley, M.M.
Cornish, Stephen
Costa-Rodrigues, João
Cotter, Joshua
Coudray, Charles
Cousins, Robert
Cozzolino, Mario
Crei, Franzcog
Crider, Krista S.
Cupisti, Adamasco
Cureton, Pamela A.
Curiel, J.A.
Curley, Robert
Curran, Christine
Custódio, Luísa
Da Graça Campos, Maria
da Silva, Robin P.
Dalen, Jeanne
Dalmas, Elise
Dalton, Caroline
D’Angelo, Stefania
Darmon, Nicole
Dasmahapatra, Asok
Dauncey, M.
Davidsson, Lena
Davis, Caroline
Davison, Glen
De Gara, Laura
De Marchi, Sergio
De Oliveira Otto, Marcia C.
De Souza, Russell
De Vries, Jeanne H.
Dean, Jeffrey A.
Decker, Eric A.
Dedoussis, George V.
Deldicque, Louise
Delgado-Calle, Jesus
Dellavalle, Diane M.
Delvin, Edgard E.
Deminice, Rafael
Denham, Bryan E.
Derave, Wim
Desai, M.
Deut, Nicolaas E.P.
Devitt, Amy A.
Dew, Tristan
Dhillon, Varinderpal
Di Iorio, Biagio
Díaz Mejía, J. Javier
Diekman, Connie
Dik, Vincent
Dirkx, Ellen
Djilali-Saiah, Idriss
Dodd, J.
Doig, Gordon
Donadio, Carlo
Donovan, Sharon M.
Doyle, Todd
Draznin, Boris
DS, Wheeler
Duarte-Salles, Talita
Ducker, Kagan
Dugan, Mike
Duh, Pin-Der
Duncan, Robin
Dunford, Elizabeth K
Duntas, Leonidas
Duttaroy, Assim
Dyerberg, Jorn
Dyson, Pamela
Eaton, S.
Edirisinghe, Indika
Efird, Jimmy
Eichler, Klaus
Ekeroma, Alec
Eknoyan, Garabed
Elmståhl, Helena
El-Sohemy, Ahmed
Enck, Paul
Eneroth, Hanna
Engle-Stone, Reina
Erceg, David
Erinosho, Temitope O.
Escanero, Jesus Fernando
Esposito, C.
Evans, David C.
Eyles, Darryl
Eyles, Darryl W.
Failla, Mark
Faith, Jeremiah
Fardet, Anthony
Fassett, Robert G
Faux, Noel
Fazeli, Pouneh
Feng, Lei
Ferguson, Lynnette
Ferguson-Stegall, Lisa
Fernandez, Maria Luz
Ferrando, Arny
Ferrer, Javier Izquierdo
Ferrie, Suzie
Field, Catherine
Fielding, Barbara
Filaire, Edith
Finamore, Alberto
Findlay, David
Finlayson, Graham
Fiorenza, Maria Teresa
Fitzgerald, Amanda
Fitzgerald, Kevin
Fitzgerald, Richard J.
Foerster, Jana
Foo, Leng Huat
Foreyt, John
Forhead, Alison J.
Fort, Patrice
Foster, J.A.
Foster, Meika
Franci, David S.
Frazier, Elisabeth
Freedman, Marjorie
Freisling, Heinz
Fresco, P.
Friedman, Jacob E.
Friesen, Carol
Fu, Tzu-Fun
Fu, Zhengwei
Fuglestad, Anita J.
Fukami, Kei
Fukazawa, Masanori
Fukuda, Michio
Fukuda, Mitsuru
Funato, Hiromasa
Funtikova, Anna
Furtado, Kelly
Gadella, Bart M.
Gagnon, Dominique D.
Gahete, Manuel D.
Galderisi, Umberto
Gallaher, Daniel D
Gallo, Valentina
Garcia, Diego
Garcia-Larsen, Vanessa
Garden, Frances
Gardiner, Paula M.
Gareau, Mélanie G.
Garinis, George
Gatterer, Hannes
Gearhardt, Ashley N.
Geelen, Anouk
Gérard, Philippe
Ghadimi, Darab
Ghosh, Rita
Giannessi, Daniela
Gibson, Deanna
Gibson, Peter R
Gill, Chris Ir
Giltay, Erik
Giovannini, Claudio
Giudetti, Anna
Glickman, Ellen
Glocker, Erik-Oliver
Gogorcena, Yolanda
Going, Scott B.
Gombart, Adrian
Gómez-Ambrosi, Javier
Gómez-Gómez, Lourdes
Gong, Jian
González-Navarro, Herminia
Goodwin, Huw
Gormaz, Juan G.
Graham, Dan J.
Grant, Ross S.
Grant, William
Grassi, Davide
Greatwood, H.C.
Grebenstein, Patricia E.
Greco, Luigi
Green, Tim
Grieger, Jessica
Griffin, Ian
Grimshaw, Kate E.C.
Gross, Micah
Grundmann, Oliver
Guasch, Marta
Gueimonde, Miguel
Guerzoni, Maria Elisabetta
Guillem, Karine
Gupta, Sanjay
Haase, Hajo
Hafeez, Bilal Bin
Hafekost, Katherine
Hagobian, Todd
Halade, Ganesh
Hamden, Khaled
Hammond, Billy
Han, Dongmei
Han, Sung Nim
Hanhineva, Kati
Hanna, Katherine
Haque, Azizul
Harding, Scott
Harland, Janice
Haroutounian, Serkos A.
Harrington, Jennifer
Harris, Cristen
Harris, William
Harris, William S.
Harrison, Fiona
Hart, Prue
Hartshorn, Kevan
Hasegawa, Daisuke
Haskell, Crystal
Hasty, Dr. Alyssa H.
Hattori, Satoshi
Haugen, Margaretha
Hauner, Hans
Hawley, John A.
Hay, William
He, Tao
Heaney, Robert
Heber, David
Hector, Debra
Hegazi, Refaat A.
Heine, Gunnar H.
Heinemann, Akos
Heinrich, Ulrike
Hellhammer, Juliane
Hemila, Harri
Hemsworth, Jaimie
Hennessy, Áine
Hennigar, Steve
Hinckson, Erica
Hipgrave, David B.
Hixson, Stefanie M.
Hobin, Erin
Hocquette, Jean-François
Hoffman, Sarah R.
Hoffmann, Kristina
Hokama, Tomiko
Holick, Michael F.
Hollis, Bruce
Holvik, Kristin
Hong, Eock Kee
Hong, Mee Young
Hook, Ingrid L.I.
Hooshmand, Shirin
Hopkins, Will
Houghton, C.A.
Houghton, Lisa A.
Houston, Mark C.
Hruby, Adela
Hsing, Chung-Hsi
Huang, Tsui-Chin
Huang, Yi-Chia
Hudson, André O.
Hughes, David
Hugo Teixeira, Vitor
Hulthén, Lena
Hummel, Sandra
Hung, Kuan-Yu
Hunter, J.O.
Hurst, Jeffrey
Husby, Steffen
Hutchins, Andrea
Hyde, Matthew
Ibiebele, Torukiri
Illner, A.
Imamura, Hiroyuki
Imperatori, Claudio
Iriti, Marcelo
Islam, M. Munirul
Itoh, Y.
Itsiopoulos, Catherine
Ivey, Kerry L.
Iwasaki, Masanori
Jaacks, Lindsay M.
Jackson, Phillipa
Jakubowicz, Daniela
Järvinen, Tero
Jean-Marc Tadié, Jean-Marc
Jeewon, Rajesh
Jeffery, Ian
Jeffry, Paul
Jiménez-Chillarón, Josep C.
Johner, Simone A.
Johnson, Ian
Johnson, Rachel
Johnston, Carol
Johnston, Craig
Jones, Andrew M.
Jones, Julie
Joosten, Etienne
Jorde, Rolf
Joven, Jorge
Joy, Edward J.M.
Ju, Sang-Yhun
Judd, Suzanne
Jun, Dae Won
Juturu, Vijaya
Kaciroti, Niko
Kahn, Richard
Kajiemail, Hiroshi
Kaklamani, Virginia
Kalliomäki, Marko
Kamada, Nobuhiko
Kamphuis, Patrick
Kane, Maureen A.
Kang, Sinyoung
Kano, Mitsuyoshi
Kanoski, Scott E.
Kaplan, Birsen
Kaplan, Bonnie J.
Karin, Amrein
Karlic, Heidrun
Karpinski, Christine
Karras, S.N.
Karunasinghe, Nishi
Kasperzyk, Julie L.
Kaster, Manuella
Kataoka, Yosky
Katayama, Shigeru
Kattelmann, Kendra
Katzberg, Hans Dieter
Kaunitz, Jonathan D.
Kavouras, Stavros
Kawakami, Shinpei
Kay, Colin
Kaye, Elizabeth Krall
Keast, Russell
Keim, N.L.
Kelley, Darshan
Kelley, Darshan S.
Kelley, Katherine
Kelley, Melissa D.
Kennedy, Deborah A.
Kenny, Anne
Kenyon, Nicholas J.
Kern, Benjamin
Key, Timothy J
Khalafallah, Alhossain A.
Khanal, Vishnu
Khymenets, Olha
Kido, Teruhiko
Kim, Byeong Mo
Kim, Cristina
Kim, Hail
Kim, Jongkyung
Kim, Mina K.
Kim, Tack-Joong
Kim, Young-jae
Kim, Yun-bae
Kingsley, Michael
Kirkpatrick, Sharon I.
Kitatani, Kazuyuki
Kłęk, Stanisław
knaapila, Antti
Knackstedt, Lori A.
Knechtle, Beat
Kniefel, Wolfgang
Ko, Hyun-Jeong
Ko, Kam Ming
Koehler, Peter
Koh, Timothy
Kojima, Masayasu
Kojo, Shosuke
Kolovou, Genovefa
Kongkachuichai, Ratchanee
Koolen, Hector Henrique Ferreira
Koot, Bart G.P.
Kopecky, Jan
Kordas, Katarzyna
Kotzka, Jorg
Kovacevic, Zaklina
Krämer, Klaus
Krebs, Nancy
Kreichauf, Susanne
Kreider, Richard
Kroeker, Karen I.
Kubis, Hans-Peter
Kuhnle, G.G.C.
Kumar, Matam Vijay
Kumar, Yogesh
Kundur, Avinash Reddy
Kuo, Shiu-Ming
Kurokawa, Masahiko
Kussmann, Martin
Kwak, Chung Shil
Kwon, Oran
Lacaille-Dubois, Marie-Aleth
Lacan, Dominique
LaFleur, Susanne
Laguna, Juan C.
Lam, Yan Y.
Lambert, Jennifer
Lanaspa, Miguel A.
Land, Mary-Anne
Lanigan, Julie
Lanou, Amy Joy
Lanthier, Nicolas
Laplante, Mathieu
Larson-Meyer, Enette
Lassek, William
Latunde-Dada, Gladys O
Laughlin, Maren R.
Laverty, Anthony A.
Lawton, Clare L.
Leclerc, Marion
Lee, Chia-Lun
Lee, Chung-Yung Jetty
Lee, Hyeon Yong
Lee, Ki Won
Lee, Kwang Ho
Lee, Myoungsook
Lee, Terence Kin-Wah
Leech, Rebecca M.
Leem, Kang-Hyun
Lei, Xin-Gen
Lemieux, Simone
Lennox, Alison
Lent, Michelle
Lentjes, Dr. Marleen
Lerner, Ulf
Leung, Ping-Chung
Levy, Yishai
Lewis, James D.
Li, Bin
Liem, Gie
Lillioja, Stephen
Lin, Ding-bo
Lin, Jin-Ding
Lin, Ping-Ting
Lionetti, Elena
Little, Jonathan P.
Liu, Biing-Hui
Liu, Peggy
Locatelli, Francesco
Lodemann, U.
Lodge, John
Lonardo, Amedeo
Longo, L.D.
López-Carballo, Gracia
Lophatananon, Artitaya
Loprinzi, Paul
Lord, Janet
Lorentz, Axel
Lorenz, Mario
Lorenzi, Rodrigo
Lorini, Dott.ssa C.
Louboutin, Jean-pierre
Lu, Chen
Lu, Jing
luca, Busetto
Lukowski, Angela F.
Lusis, Aldons J.
Lutsey, Pamela L.
Luyckx, Valerie A.
Ma, David W.L.
Ma, Jin Yeul
Macdiarmid, Jennie
Macdonald, Jamie Hugo
MacFarlane, Amanda J.
Mackenzie, Lincoln
Macpherson, Helen
Madar, Zecharia
Madej, Dawid
Maes, Michael
Magann, Everett F.
Maggini, Silvia
Maggio, Albino
Magnusson, Roger
Maher, Timothy J.
Mair, Christiane
Maitland, Kathryn
Makarenko, Vladislav
Mallard, Simonette R.
Manco, Marco
Mangialasche, Francesca
Mann, Samuel J.
Mantovani, Giovanni
Marakis, Georgios
Marek, Ryan J.
Marian, Mary
Mariotti, François
Markhus, Maria Wik
Martel, Fátima
Martínez González, Miguel Ángel
Martínez-Cuesta, M. Carmen
Martínez-Micaelo, Neus
Maruvada, Padma
Mas, Emilie
Maslova, Ekaterina
Masulli, Maria
Matsumoto, Ippei
Matsumoto, K.
Matsumura, Kenta
Matsuoka, Yutaka
Mattar, Melanie
Mattijssen, Frits
McArthur, Jennifer O.
McCarthy, David
McCarthy, John
McCarty, Catherine A.
McCarty, Mark
Mcclung, James
McConell, Glenn
McDaniel, John
Mcdougall, G.J.
McDougall, John
McGrath, John J.
McGrath, Kristine
McIntosh, Graeme
McKenna, Malachi J.
McManus, Alexandra
McQuestion, Maurene
Mecocci, Patrizia
Medlock, Amy
Mehta, Rajendra G.
Meijers, Joost C.M.
Mekhedov, Sergei
Meli, Rosaria
Memon, M.A.
Mencarelli, Fabio
Mennella, Julie
Meredith, M. Elizabeth
Messner, Donald J.
Meyer, Anne
Michels, Alexander J.
Miguel, M.G.
Miller, Joshua W.
Miller, Gary D.
Miller, Matt
Miller, Matthew
Miller, Tracie L.
Miller, Woodgate
Milte, Catherine
Minutolo, Roberto
Miraglia, Emanuele
Mischley, Laurie
Mishra, Suman
Mishur, Robert J.
Mo, Huanbiao
Moffat, Tina
monro, John.
Moody, A. John
Moon, Jordan
Moore, Amanda
Morales, Eva
Morales-González, José
Moran, Carthage P.
Morelli, Lorenzo
Moretti, Diego
Moriyama, Tatsuya
Morris, Howard
Morteza, Afsaneh
Mounier, Catherine
Mountjoy, Margo
Muhlhausler, Beverly
Mukai, Yuuka
Mukhar, Hasan
Müller, W.E.G.
Mulligan, Angela A
Mullin, James M.
Munoz, Colleen
Murakami, Haruka
Murff, Harvey J.
Mustacich, Debbie J.
Mutti, Elena
Mwatsama, Modi
Nagaoka, Satoshi
Nagineni, Chandrasekharam N.
Nair, Krishnapillai Madhavan
Nairz, Manfred
Nakahama, Kenichi
Nammi, Shrinivas
Naska, Androniki
Navarro, Carlos Gonzalez
Nelson, Michael
Netticadan, Thomas
Neu, Josef
Neumann, Charlotte
Newsholme, Philip
Neyraud, Eric
Nguyen, Douglas L.
Nicoletti, Claudio
Nieman, David C.
Nievar, M. Angela
Nijdam, Durk
Niki, Etsuo
Nikolaidis, Michalis G.
Nino, Russo
Nissim, Ahuva
Nosova, Emily
Nwaru, Bright
Oba, Shino
Oberhelman, Sara
Obrenovich, Mark E.
O'Connell, Thomas
O’connor, Helen T.
Oda, Hiroaki
O’Dell, Sandra
Oelkrug, Christopher
Oesterreicher, Christoph
Ogata, Tadanori
Oglesby-Sherrouse, Amanda
Oinonen, Kirsten A.
Ojeda, N.B.
O'Keefe, Stephen J.D.
Olefsky, Jerrold M.
Olek, Rovert A.
Onakpoya, Igho J.
Onda, Ken
O'neil, Carol
Ormsbee, Michael
Ornish, Dean
Osher, Yamima
O'Sullivan, Aifric
Oterhals, Åge
Pacanowski, Carly
Pacetti, Deborah
Pachon, Helena
Pacilli, Maurizio
Page, Amanda J.
Paiva-Martins, Fátima
Pala, Valeria
Panahi, Shirin
Pantano, Kathleen Joan
Paoli, Antonio
Papadakis, Sophia
Papadimitriuou, Konstantinos
Papadopoulou, Eleni
Papetti, Adele
Park, Hwa-Jin
Park, Kyong
Park, Yongsoon
Parletta, Natalie
Parthasarathy, Sampath
Patten, Glen S.
Patterson, Bruce
Patterson, Michael
Payne, Martha
Peairs, Abigail D.
Peake, Jonathan
Pedram, Pardis
Peñagaricano, Francisco
Peng, Guang
Pepino, M. Yanina
Perks, C.M.
Peter Stehle, Peter
Péterfy, Miklós
Peterson, Catherine A.
Peterson, Julia
Peterson, Linda
Pezzarossa, Beatrice
Phillips, Stuart M.
Pinto, John T.
Pittman, Karin
Plösch, Torsten
Plourde, Mélanie
Poblaciones, Maria J.
Polidori, Maria Cristina
Pothos, Emmanuel N.
Pottala, James
Powers, Hilary J.
Powles, John W.
Pradelli, Lorenzo
Premkumar, Muralidhar
Pritchard, Janet
Privitera, Gregory
Qi, Lu
Qin, Bolin
Qu, Yan
Quatromoni, Paula
Raatz, Susan K.
Rachek, Lyudmila
Radmacher, Paula
Ragaee, S.M.
Raha, Sandeep
Raj, Sudha
Ranadheera, C. Senaka
Randall, Philip
Randall-Simpson, Janis
Rasmussen, Nicolas
Rasperini, Giulio
Rattan, Sureshi. S.
Rauch, Bernhard
Reed, Bruce
Rehm, Colin
Reid, Gregor
Reimer, Raylene A.
Reis, Joanna
Reis, Mariza G.
Remacha, A.F.
Rezamand, Pedram
Rigottier-Gois, Lionel
Rhodes, Jonathan
Ridoutt, Brad
Rijntjes, Eddy
Riley, Malcolm D.
Riverin, Bruno
Roa Romero, Laura M.
Roberts, Michael
Robertson, Graham
Roche, M.
Rodriguez, Ana
Rodriguez-Rodriguez, Rosalia
Rogers, Lynette K.
Rombouts, Ine
Romero, Maria-Paz
Rondanelli, Mariangela
Root, Martin
Rosa, Angelo
Rose, Devin
Rosendale, Douglas Ian
Ross, A. Catherine
Ross, Alastair B.
Rossi, Mauro
Rothers, Janet
Rousseau, Anne-Sophie
Rousseau, G.
Rozowski, Jaime
Rucklidge, Julia
Rudkowska, Iwona
Rurak, Dan
Rush, Elaine
Rust, Rosanne
Ruxto, Carrie
Ryan, Lisa
Rybalka, Emma
Ryu, Jae-Ha
Sachan, Dileep S.
Saheki, Takeyori
Sakuma, Kunihiro
Sale, Craig
Salmerón, Ivan
Salvini, Filippo
Samocha-Bonet, Dorit
Sanchez-Muniz, Francisco
Sandoval, Claudio
Sangild, Per T.
Santini, Antonello
Sapuntzakis, Maria
Sartore, Giovanni
Sasazuki, S.
Satarug, Soisungwan
Sathishkumar, K.
Sato, Kenji
Sato, Shin
Satta, Yoko
Savica, Vicenzo
Saw, Constance Lay Lay
Schiavone, Stefania
Schini-Kerth, Valérie
Schlienger, Jean-Louis
Schmidt-Heydt, Markus
Schoeler, Natasha E.
Schroeder, Helmut
Schubert, Matthew M.
Schultz, Clyde L.
Schulze, Kerry
Schupp, Michael
Schweitzer, Dietrich
Scoffield, Jessica
Scott, Dr. Hayley
Scragg, Robert
Sellke, Frank W.
Seluanov, Andrei
Senchina, David
Seo, Hyung Seok
Serio, Francesco
Serra, Carlo
Serra, Serra
Serralheiro, Maria Luísa M.
Serra-Majem, Lluís
Serrano, María
Serrat, Maria A.
Seth, Anju
Shahab-Ferdows, Setti
Shakur, Yaseer A
Sharoni, Yoav
Shen, Chwan-Li
Shertzer, Howard G.
Shi, Zumin
Shiloach, Joseph
Shimizu, Takahiko
Shiva, Sruti
Shoben, Abigail B.
Shrapnel, Bill
Siafarikas, Aris
Siddappa, Asha Jyothi
Silver, Heidi J.
Simeonov, V.
Simopoulos, Artemis
Siniscalco, Dario
Sioen, Isabelle
Sjögren, Per
Skinnert, Tina
Skipworth, Richard J.E.
Skov, Laurits
Smedslund, Geir
Smith, Adam
Smith, Caren E.
Smith, E.
Smith, Kylie J.
Smith, Lindsey
Sørensen, L.B.
Specker, Bonny
Spedding, Simon
Srzednicki, George
Stachowicz-Stencel, Teresa
Staege, Martin S.
Staiano, Anna-Maria
Stanhope, Kimber
Stefanidou, Maria E.
Stefanska, Barbara
Stefanutti, Claudia
Steinberg, Francene M.
Steinle, Nanette I.
Stephen, Mary C.
Sterkowicz, Stanislaw
Stevenson, D.E.
Stilli, Donatella
Stone, William
Strain, Sean
Strandvik, Birgitta
Strauss, William
Su, Hui-Min
Su, Xiao
Subramanian, Veedamali S.
Sugatani, Junko
Sugimoto, Ken
Sugiyama, Kemmyo
Suh, Miyoung
Sun, Guang
Sun, Hui Sun
Sung, Ping-Jyun
Szeto, Yim Tong
Takahisa, Kanekiyo
Takeda, Norifumi
Tako, Elad
Tamura, Tsunenobu
Tan, S.-Y.
Tanaka, Hiroyuki
Tanofsky-Kraff, Marian
Tappia, Paramjit S.
Tappy, Luc
Tashiro, Hirotaka
Taylor, Lem
Teas, Jane
Temple, Jennifer L.
Tepper, Beverly J.
Terai, Shuji
Terrin, Gianluca
Tessitore, Antonio
Thelen, Paul
Thèvenod, Frank
Thoma, Christian
Thomas, David Travis
Thomas, Lynn K.
Thunders, Michelle
Tibolla, Gianpaolo
Tiidus, Peter M.
Timmerman, Kyle
Tlaslakova-Hogenova, Helena
Tobwala, Shakila
Tomofuji, Takaaki
Torheim, Liv Elin
Tosi, Paola
Tou, J.C.
Tran, Minh-Ha
Transler, Catherine
Travagli, Valter
Trayhurn, Paul
Tremblay, Angelo
Triantafillidis, John K.
Trosko, James E.
Tsai, Ying Chieh
Tsao, Po-Nien
Tscholl, Philippe
Tsiplakou, E.
Tsoli, Maria
Tsuji, Petra
Tulchinsky, Ted
Túnez, Isaac
Turon, X.
Tussing-Humphreys, Lisa
Twells, L.
Ufer, Christoph
Ulatowski, Lynn
Um, Sung Hee
Uribarri, Jaime
Ute, Alexy
Uto, Takuhiro
Utzschneider, Kristina M.
Vaisburg, Arkadii
Vajro, Pietro
Valdivielso, P.
Valent, Francesca
Valenti, Luca
Valerio, Costa
Vallgårda, Signild
van Baak, Marleen A.
van Ballegooijen, Hanne
van Heerden, P.V.
van Rossem, Lenie
van Schoor, Natasja M.
Vandenplas, Yvan
Vandewoude, Maurits F.J.
Vapaatalo, Heikki
Varela-Moreiras, G.
Vargas, Carmen Regla
Vecchia, Carlo
Vecoli, Cecilia
Vega, Renato S.A.
Velloso, Licio A.
Venn, Bernard
Verbeke, Kristin
Verduci, Elvira
Vermaak, Ilze
Vicente, Laura
Vickers, Mark
Vieira, A.
Vinciguerra, Manlio
Visavadiya, Nishant P.
Volterman, Kim
Von Hurst, Pamela
von Lintig, Johannes
von Schacky, Clemens
von Wright, Atte
Wada, Jun
Wagner, Carol
Wagner, Erwin F.
Wagstaff, Carol
Wai, AU-YEUNG Kathy Ka
Walker, Alan W.
Walker, Celia G.
Wang, Chi Chiu
Wang, Gene-Jack
Wang, Hui
Wang, Jiawei
Wang, Lu
Wang, Peizhong Peter
Wang, Piwen
Wang, Ting
Wang, Xiaohui
Wang, Ying
Wang, Zhanxiang
Ward, Leigh
Ward, Mary
Wasse, Lucy
Waterhous, Therese S.
Watkins, Adam J.
Watkins, Richard R.
Wauquier, Fabien
Weaver, Connie
Weaver, Ian
Webb, Andrew J.
Webster, Jacqui
Weed, Harrison
Weinberg, Gene
Welsh, Jean
Westerterp, Klaas
Westerterp, Klaas R.
Whelan, Jay
Whisner, Corrie
Whisner, Corrie M.
Whiting, Susan
Widen, Elizabeth
Wijnkoop, Lenoir
Wilder-Smith, C.H.
Wilkinson, J.M.
Williams, Anne
Williams, John
Williams, Nancy I.
Williamson, Patricia
Wilson, Gabriel
Winter, Sebastian
Wlazlo, Nick
Włodarek, Dariusz
Wong, Sze Choong
Woo, Jessica Graus
Worsley, Tony
Wu, Jian-Ping
Xian, Cory J.
Xing, Mingzhao
Xu, Fenglian
Yakub, Mohsin
Yamamoto, Hironori
Yang, Chung S.
Yang, Daichang
Yang, Mhan-Pyo
Yang, R.-H.
Ye, Sang-kyu
Yeh, Shu-Lan
Yeung, Edwina
Ying, Danyang
Yokoyama, Yoshihito
Yu, Danxia
Yun, Jong Won
Yung, Raymond
Yurko-Mauro, Karin
Zamora-Ros, Raul
Zapolska-Downar, D.
Zara, Vincenzo
Zarrelli, Armando
Zhang, Guirong
Zhang, Xuehong
Zhao, Liang
Zhao, Yuanxiang
Zhao, Yuejen
Zhou, Fang Qiang
Zhou, Yifa
Zhu, Meijun
Ziouzenkova, Ouliana
Zittermann, Armin
Zlokovic, Berislav
Zwart, Sara

